# The association between cholesterol efflux capacity and apolipoprotein A1: systematic review and meta-analysis

**DOI:** 10.11613/BM.2025.030506

**Published:** 2025-10-15

**Authors:** Linas Černiauskas, Eglė Mazgelytė, Dovilė Karčiauskaitė

**Affiliations:** 1 Department of Physiology, Biochemistry, Microbiology and Laboratory Medicine, Institute of Biomedical Sciences, Faculty of Medicine, Vilnius University, Vilnius, Lithuania; 2 Biomarker Research Laboratory, Institute of Translational Health Research, Faculty of Medicine, Vilnius University, Vilnius, Lithuania

**Keywords:** apolipoprotein A1, cholesterol efflux, reverse cholesterol transport, meta-analysis, systematic review

## Abstract

**Introduction:**

High-density lipoprotein (HDL) particles are key participants in reverse cholesterol transport. Cholesterol efflux capacity (CEC) and apolipoprotein A1 (Apo A1) are HDL-related biomarkers often used to evaluate HDL particle functionality and quantity. This study aimed to assess the correlation between CEC and Apo A1 concentrations and explore whether methodological aspects influence the correlation results.

**Materials and methods:**

This meta-analysis was prospectively registered in the PROSPERO database (registration number CRD42024552535). Three databases, PubMed, Web of Science, and Cochrane Library, were screened for the studies published between January 2000 and May 2024. The correlation results were analyzed using a random-effects model, and sensitivity and subgroup analyses were performed.

**Results:**

A total of 19 studies with 4967 participants were included. This meta-analysis’s results indicated a statistically significant positive moderate strength correlation between CEC and Apo A1 concentrations. A high level of study heterogeneity was observed among the included studies. Further exploration into this heterogeneity revealed that different cell culture lines and cholesterol acceptors used to evaluate CEC impact the overall result of the pooled correlation estimate. The methods used to evaluate Apo A1 did not significantly affect the correlation estimate between CEC and Apo A1 concentrations.

**Conclusions:**

The correlation between CEC and Apo A1 lacks strength and consistency for Apo A1 being used as a surrogate marker for HDL function in a clinical setting. Currently, there is a high need for the standardization of CEC measurement methodologies that impact the overall results and comparability of the studies that have already been conducted.

## Graphical abstract

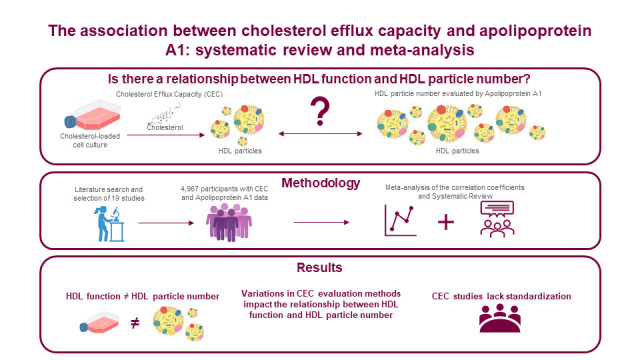

## Highlights

• There is a positive moderate strength correlation between cholesterol efflux capacity and apolipoprotein A1

• The cell line and the cholesterol acceptor affect the correlation

• In contrast, the apolipoprotein A1 evaluation method did not affect the correlation

• Cholesterol efflux capacity assays must be standardized due to their high variability

## Introduction

High-density lipoprotein (HDL) particles participate in reverse cholesterol transport (RCT), during which cholesterol is acquired from peripheral tissues and transported into the liver for removal from the body (*1*). Physiologically, this process is crucial for removing excess cholesterol from macrophages in arterial walls to prevent or reduce the formation of foam cells and is proposed to slow the progression of atherosclerosis (*2*).

The first step in RCT is cellular cholesterol efflux, and it occurs *via* four main pathways: aqueous diffusion, scavenger receptor class B type 1 (SR-B1), ATP-binding cassette transporter G1 (ABCG1), and ATP-binding cassette transporter A1 (ABCA1). Aqueous diffusion and SR-B1 pathways are passive cellular cholesterol transport, while ABCG1 and ABCA1 are active transport, requiring ATP to occur. Most of the cellular cholesterol efflux is attributed to the active transport by the ABCA1 pathway (*3*, *4*). This pathway requires ABCA1 association with the main HDL protein Apo A1, making Apo A1 a crucial molecule in the first step of RCT (*4*, *5*).

The key laboratory measure of cellular cholesterol transport is cholesterol efflux capacity (CEC), which allows for *in vitro* evaluation of the cellular cholesterol efflux from macrophages or other cell types into the acceptor containing HDL particles using specifically labeled cholesterol molecules (*6*). However, this method is very complex, time- and resource-consuming, and lacks standardization, which reduces its reproducibility and accessibility in clinical settings. On the other hand, Apo A1 is easily measurable using immunoassays and allows the estimation of HDL particle numbers (*7*). Despite the biological connection between Apo A1 and CEC, the strength and consistency of their relationship remain a topic of debate. Measurement of Apo A1 concentration does not fully capture the functional properties of HDL particles, which may be better reflected by direct assessment of CEC. Individual studies have reported varying degrees of correlation, influenced by factors such as assay methodologies, population heterogeneity, and disease states. A better understanding of the relationship between Apo A1 concentrations and CEC is critical for evaluating the utility of Apo A1 as a surrogate marker for HDL functionality and its relevance in predicting cardiovascular disease (CVD) risk.

The aim of this systematic review and meta-analysis was to evaluate the correlation between two HDL-related laboratory biomarkers - CEC, which denotes HDL function, and Apo A1, which denotes HDL particle number, and identify whether different methodological aspects involved in CEC and Apo A1 evaluation affect the correlation results.

## Materials and methods

### Search strategy and selection criteria

This study was performed according to the guidelines of the Preferred Reporting Items for Systematic Reviews and Meta-Analyses (PRISMA) statement (*8*). We conducted a systematic literature search in the PubMed, Web of Science, and Cochrane Library databases for articles describing the association between Apo A-I concentrations in blood serum or plasma and CEC (Prospero CRD42024552535). The articles were published from 1 January 2000 to 1 May 2024. In PubMed and Web of Science databases, the following keywords and phrases were used as search terms: “apolipoprotein a 1”, “Apolipoprotein A I”, “Apo A-I”, “Apo A1”, “Apolipoprotein A1”, “ApoA-1”, “ApoA-I”, “Apolipoprotein A-1”, “Apolipoprotein A 1”, “Apolipoprotein A1”, “Apo A-1”, “Apo A1” “cholesterol efflux capacity”. In the Cochrane Library database, the following combination of terms “cholesterol efflux capacity“ AND “human” was used. The inclusion criteria were based on the PICO framework. They included: Population (*1*) the study was a clinical trial or other type of study (a cross-sectional, a case-control or a cohort study) including healthy or apparently healthy adult subjects; Intervention (*2*) studies that evaluated Apo A1 concentrations in blood serum or plasma; Comparator (*3*) studies that evaluated CEC as the variable correlated with Apo A1; Outcome (*4*) studies reporting a correlation coefficient (r) or it could be calculated from the coefficient of determination; Other filters (*5*) studies written in English. The exclusion criteria included (*1*) studies conducted in animal models and (*2*) studies with clinical interventions (pharmacological, instrumental, surgical) affecting lipid metabolism.

The reference lists of retrieved articles were also checked for relevant articles. Search results were exported, and duplicates were removed before screening. Full search strategies for all databases are included in Supplemental table S1.

### Assessment of the quality of the studies

One self-derived assessment tool was used to evaluate the quality of the included studies. The study quality was evaluated by selecting seven criteria relevant to the methodologies of the included studies and the field of clinical chemistry and laboratory medicine. The tool included seven criteria across these fields: study design, sample size, biomarker measurement, blinding of laboratory analysis, confounding factors, and statistical analysis (Supplemental table S2). The selection included the following criteria: the study design is appropriate for correlation analysis (D1), the sample size is justified or statistically powered for detecting biomarker correlation (D2), Apo A1 was measured using a clinically validated method (D3), CEC was assessed using a clearly described and reproducible experimental method (D4), biomarker measurements were blinded to clinical data or outcome (D5), potential confounders identified and controlled for (D6), an appropriate statistical method was chosen and used (D7). Each criterion received a grade from 0 to 2 points. The tool devises an overall score from 0 to 14. The studies with an overall score of 0 to 6 were considered low quality, 7 to 10 were considered moderate quality, and the studies with an overall score of 11 to 14 were considered high quality. Two authors (L.Č. and E.M.) independently evaluated the quality of the included studies. The disagreements between the study evaluation of the two authors were further discussed with the involvement of the third author (D.K.), and a consensus was reached.

### Data extraction

The data was extracted independently by two authors (L.Č. and E.M.) from the included studies, and a table was devised to provide information on the author(s), year of publication, type of the study, total sample size, sample size included in the analysis, sex, cholesterol label, measurement method of Apo A1, cholesterol acceptor, cell line and correlation coefficient. For the studies where the correlation coefficient was not available in the text, but the information was provided on its calculation, the correlation coefficient was manually calculated from the coefficient of determination. In cases where two correlation coefficients were provided using different cell lines and/or methods, the correlation coefficient devised from total CEC values or the monocyte-macrophage lineage cell line was used to avoid duplicating the data from the same study sample.

### Statistical analysis

Correlation coefficient r values ranged from - 1 to 1 and were used as the effect size measures. The Fisher‘s Z logarithmic transformation was used to normalize the variance, eliminate bias, and calculate the Fisher‘s Z scores. Fisher‘s Z scores were used to estimate the effect sizes and their confidence intervals. The sample sizes n were used to weight the contributions of each study to the overall result. The Fisher‘s Z scores were transformed back to correlation coefficients for easier interpretation and visualization. The Chi^2^ (Cochran‘s Q) and *I^2^* tests were used to evaluate the heterogeneity among the included studies. An *I^2^* statistic of 25% indicated low, 50% moderate and 75% high heterogeneity. For the article’s clarity and visual representation, Chi^2^ was used throughout the text and images instead of Cochran‘s Q. The random effects model was used if *I^2^* statistic was ≥ 50% and P ≥ 0.10 across studies, indicating statistically significant moderate to high heterogeneity. A fixed effect model was used if the *I^2^* statistic was < 50% and P < 0.10 across studies. The prediction interval was calculated to assess the range of true effects in future studies, which was assumed to be broader than the confidence interval when there is high heterogeneity among studies. Subgroup analyses were performed to evaluate the possible sources of heterogeneity based on cell line, cholesterol acceptor, and the method used for Apo A1 evaluation. Sensitivity analyses using the leave-one-out strategy were performed to evaluate the robustness of the analysis results. To assess the risk of publication bias contour-enhanced funnel plots were used for the visual evaluation of the data and Egger’s and Begg’s tests were performed. For statistical significance P < 0.05 was set as a threshold. The meta-analysis was conducted using R Statistical Software (v4.3.3; R Core Team 2024).

## Results

### Study identification and selection

**Figure 1:**
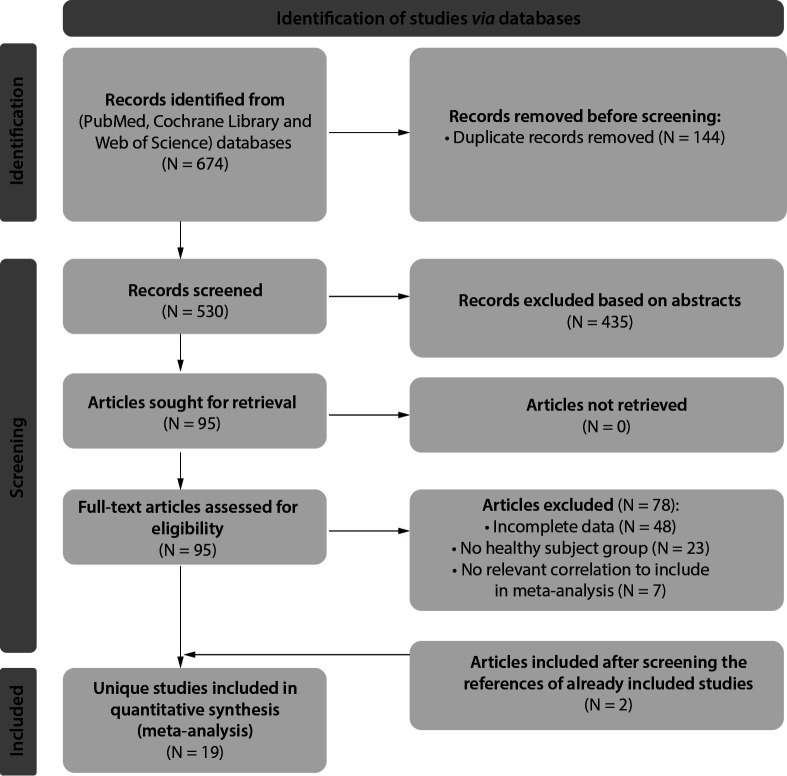
Flow chart of the literature search

In total, 674 studies were identified by searching the databases. The 144 study duplicates were removed from the initial list, and 530 studies remained to screen the abstracts. Abstract screening allowed the exclusion of 435 studies, with 95 studies remaining for retrieval. Full-text articles from all 95 studies were retrieved and assessed for eligibility. The assessment removed 78 studies: 48 due to incomplete data, 23 had no healthy subject group, and seven did not report relevant correlations between CEC and Apo A1 to include in the meta-analysis. A total of 17 studies remained after the assessment, and two more studies were included after a thorough review and additional screening of the references for the already included studies. Overall, 19 studies reporting relevant correlates between CEC and Apo A1 were included in this review and meta-analysis, with 4967 participants (Figure 1).

### Study characteristics

Of the 19 studies included in the meta-analysis the most were from the USA (N = 5), China (N = 3), Iran (N = 3), and the Netherlands (N = 2) (*9*-*21*). The remaining six studies were from Australia (N = 1), Canada (N = 1), Czech Republic (N = 1), France (N = 1), India (N = 1), and Sweden (N = 1) (*22*-*27*). One study (N = 20) did not include information on the sex of the control group (*25*). Therefore, acknowledging that this small sample size does not significantly affect the overall sex distribution of the sample, the estimated percentage of male sex participants in the sample used in this meta-analysis was 63.1%. Three studies included only male participants, while one study included only female participants (*18*, *20*, *22*, *24*).

Overall, high variability was observed in the choice of cell line used for CEC evaluation. Among the 19 studies included in this meta-analysis, six cell lines were identified being used to evaluate CEC: J774 murine macrophages (N = 9), THP-1 human macrophages (N = 3), Fu5AH murine hepatic cells (N = 3), human fibroblasts (N = 2), CHO Chinese hamster ovary cells (N = 1) and RAW 264.7 murine macrophages (N = 1) (*9*-*27*). Even higher variability was observed in the choice of cholesterol acceptor. Total of 8 different cholesterol acceptors were used throughout the studies: 2.8% Apo B-depleted serum (N = 6), 2% Apo B-depleted serum (N = 2), 1% Apo B-depleted serum (N = 1), not defined percentage of Apo B-depleted serum (N = 1), 2.8% Apo B-depleted plasma (N = 3), 5% plasma (N = 1), 5% serum (N = 3), 1% plasma (N = 2) (*9*-*27*). A low variability was observed between methods used for Apo A1 evaluation with only three main methods used: immunoturbidimetry (N = 7), immunonephelometry (N = 6), enzyme-linked immunosorbent assay (ELISA) (N = 3) and the method was not available (N/A) (N = 3) in some studies.

The study quality was evaluated using a self-developed assessment tool introduced in section 2.2. The evaluation results revealed that 42.1% (N = 8) of studies were considered high-quality and 57.9% (N = 11) of moderate quality according to the selected criteria (Supplemental table S3 and Supplemental figure S1).

The characteristics of the 19 studies included in the meta-analysis are summarized in Supplemental table S4.

### Correlation between cholesterol efflux capacity and Apo A1

**Figure 2:**
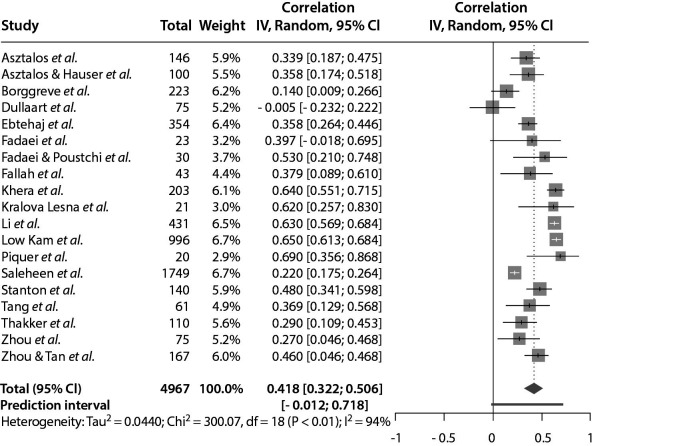
Forrest plot examining the correlation between cholesterol efflux capacity (CEC) and apolipoprotein A1 (Apo A1). The random effect model revealed that CEC and Apo A1 concentrations had a statistically significant positive moderate correlation. However, the *I^2^* and Cochran’s Q statistics indicated high heterogeneity between studies included in the model. CI - confidence interval.

The random effect model revealed that CEC and Apo A1 concentrations had a statistically significant positive moderate correlation (*r* = 0.42, 95% confidence intervals (CI): 0.32 to 0.51, *P* < 0.001). However, the *I^2^* and Cochran’s Q statistics indicated high heterogeneity between studies included in the model (*I^2^* = 94%, 95% CI: 91.9 to 95.5; Chi^2^ = 300.07, df = 18, P < 0.01) (Figure 2). In addition, the wide prediction interval (- 0.012; 0.718) ranging from weak negative to strong positive effects indicated that the results of future studies evaluating the correlation between CEC and Apo A1 may have considerable variation. The range of the calculated prediction interval further supported the notion of high heterogeneity between studies.

### The evaluation of study bias

**Figure 3:**
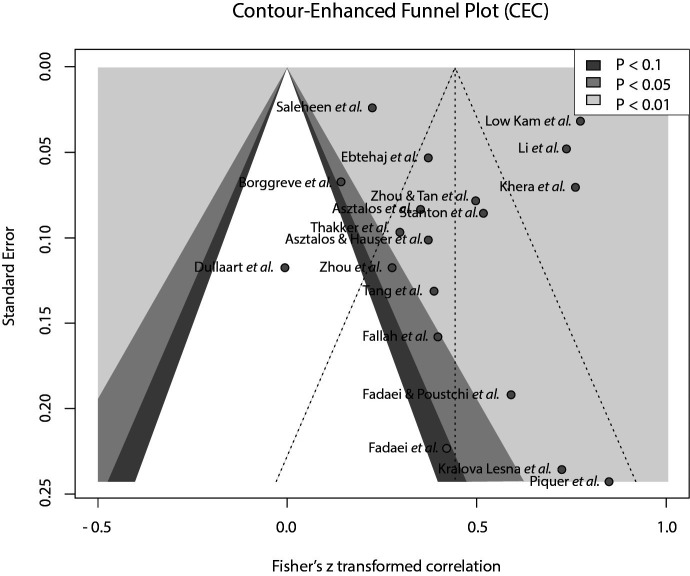
Funnel plot for publication bias. The risk of study bias was explored using visual evaluation of contour-enhanced funnel plot and quantitative evaluation by Egger’s and Begg’s test results.

The risk of study bias was explored using visual evaluation of contour-enhanced funnel plot and quantitative evaluation by Egger’s and Begg’s test results. The visual inspection of the contour-enhanced funnel plot indicated some asymmetry and that there likely was a risk of study bias (Figure 3). However, Egger’s (t = 0.21, df = 17, P = 0.836) and Begg’s test results (Z-score = 0.84, P = 0.401) revealed that there was no statistically significant evidence for publication bias among the studies included in this meta-analysis.

### Sensitivity analysis

**Figure 4:**
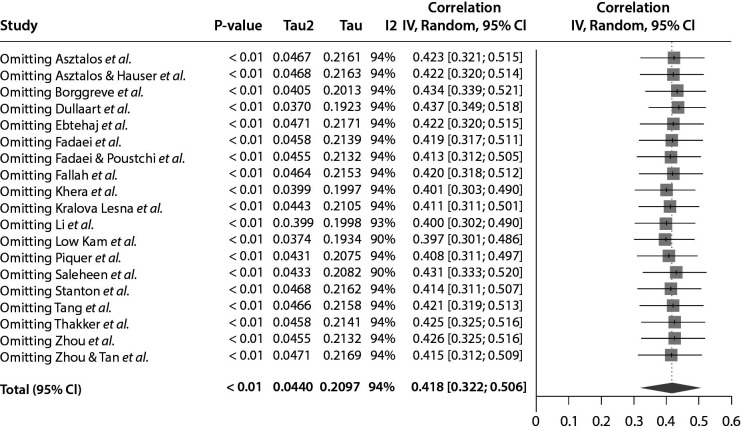
Forrest plot of sensitivity analysis done using leave-one-out method. The analysis revealed that the overall result of the pooled correlation estimate did not change by excluding any of the studies (*P* < 0.01). CI - confidence interval.

Sensitivity analysis using the leave-one-out method was conducted to assess whether the findings of meta-analysis are robust (Figure 4). The analysis revealed that the overall result of the pooled correlation estimate did not change by excluding any of the studies (P < 0.01). These findings indicated that the overall result of this meta-analysis is robust.

### Subgroup analysis

As the sensitivity analysis indicated, the robustness of the pooled estimate of the correlation between CEC and Apo A1 and the risk of bias evaluation revealed that there was likely no study bias. The causes of between-study heterogeneity were further explored. To assess the possible causes of heterogeneity among included studies, subgroup analyses based on cell line, cholesterol acceptor, and Apo A1 method were performed (Supplemental figures S2-4). Subgroup analyses revealed that cell line type (Chi^2^ = 25.22, df = 5, P < 0.01) and acceptor type (Chi^2^ = 27.44, df = 7, P < 0.01) used in the evaluation of CEC significantly affected the pooled correlation coefficient between CEC and Apo A1. The chosen method to evaluate Apo A1 (Chi^2^ = 0.14, df = 3, P = 0.99) did not significantly affect the correlation between CEC and Apo A1.

## Discussion

This meta-analysis explored and summarized the findings of previous studies with inconsistent results on CEC and Apo A1 correlation. Even though we found the pooled correlation between CEC and Apo A1 to be statistically significant, positive, and of moderate strength, the studies had high variability in their methodologies, resulting in high heterogeneity. In addition, identifying cell line and cholesterol acceptor as two factors highly varying between these studies and impacting the overall correlation between CEC and Apo A1 highlights methodological discrepancies in CEC evaluation.

The findings of this meta-analysis indicate that Apo A1 is associated with CEC, but it lacks strength and consistency for practical use in clinical settings. The misconception that high HDL concentrations indicate HDL particles have better atheroprotective qualities is widespread in clinical settings. The findings of this study, in part, demonstrate that further research is needed in the field of HDLs and their functionality, as well as that Apo A1 should not be used in clinical settings as a surrogate marker of HDL function.

Various cell lines are used for CEC evaluation in the laboratory setting. The main cell lines used in most CEC studies are monocyte-macrophage lineages, such as J774 murine macrophages, THP-1 human monocytes, and RAW 264.7 murine macrophages (*28*, *29*). The choice of these cell lines is based on the direct involvement of monocytes and macrophages in the pathophysiological process of atherosclerosis. Monocytes and macrophages are present in atherosclerotic lesions and are essential for phagocytosing the accumulated lipoproteins and lipid aggregates in the arterial wall (*30*). The use of other types of cells expressing ABCA1, such as Fu5AH murine hepatic cells, human fibroblasts, or CHO Chinese hamster ovary cells, is highly debated, as these cells are not directly involved in the physiological mechanisms of lipid phagocytosis and removal from atherosclerotic lesions. The preferred choice of cell line for CEC evaluation is monocyte-macrophage lineage cells due to their involvement *in vivo* in pathophysiological mechanisms of atherosclerosis. Despite the choice of cells for CEC experiments, using cell lines introduces high variability in cell cultivation techniques and poses a risk for contamination, genetic drift, or misidentification (*31*). Some novel cell-free assays for cholesterol efflux were developed to reduce such risks that could affect overall CEC measurement and result reproducibility (*32*, *33*). Instead of cell lines serving as cholesterol donors, these methods employ cholesterol-loaded liposomes or evaluate fluorescent-labeled cholesterol uptake by HDL particles directly from reaction media. However, these methods are yet to be compared with conventional cell-based CEC measurement methods to assess their agreement and applicability.

Another key methodological factor indicated in this study that affects the correlation between CEC and Apo A1 is the choice of cholesterol acceptor. The cholesterol acceptors vary widely between studies, ranging from whole diluted human serum or plasma to Apo B-depleted versions of the same specimens. The pioneering experiments in the field of CEC done by de la Llera Moya and colleagues revealed that most cellular cholesterol efflux is attributable to the serum fraction depleted from Apo B-containing particles (*34*). The whole serum or plasma includes all lipoprotein particles, including Apo B-containing proatherogenic lipoproteins such as very-low-density lipoproteins (VLDL), intermediate-density lipoproteins (IDL), and low-density lipoproteins (LDL). These particles exchange their lipids with Apo A1-containing HDL particles *via* the enzymatic activities of phospholipid-transfer protein (PLTP) and cholesteryl-ester transfer protein (CETP) (*35*). The overall cholesterol efflux from cells studied *in vitro* may be affected by the presence of Apo B-containing lipoprotein particles, as cholesterol effluxed to HDLs can be readily transported to these particles. This transfer of cholesteryl esters from HDL to Apo-B-containing particles was shown to occur in the same pioneering CEC study by de la Llera Moya and colleagues (*34*). Employing whole serum or plasma as cholesterol acceptors in CEC evaluation does not accurately evaluate the cholesterol efflux from cells to HDL particles, as some of the effluxed cholesterol can be further transferred to Apo B-containing lipoproteins, impacting the overall efflux measure. Apo B-depleted serum or plasma virtually contains no VLDL, IDL, or LDL particles; thus, the overall cholesterol efflux may be attributed mainly to HDL particles (*36*, *37*). This is usually the preferred method to evaluate CEC as it is regarded as a marker of HDL function. Most CEC studies use Apo B-depleted serum or plasma at varying percentages of the final efflux media with chemical precipitation methods, such as polyethylene glycol (PEG) or dextran sulfate with magnesium. These precipitation methods are used due to their easy application and quick throughput, essential in CEC studies employing large sample sizes. Other studies employ immunoaffinity separation or the ultracentrifugation method, known as the gold standard for lipoprotein separation. Each of these methods distinctly affects HDL composition and distribution, thus introducing additional factors affecting the comparability of CEC measurement studies (*36*). In addition, recently discovered extracellular vesicles (EVs) were shown to co-isolate with HDL particles using traditional isolation methods (*38*). Extracellular vesicles have also been shown to participate in cholesterol metabolism (*39*). Virtually none of the current CEC studies evaluated the involvement of EVs in cholesterol efflux. Therefore, an additional separation step, using size exclusion chromatography (SEC) or other relevant methods, is highly recommended (*40*). According to the current studies, the most suitable acceptor for CEC evaluation is Apo B-depleted serum or plasma, as it allows for the direct evaluation of only HDL-dependent cholesterol efflux. However, further isolation techniques should be employed to remove EVs that may interfere with the net cholesterol efflux.

The results of this study indicate that the chosen Apo A1 evaluation methodology does not impact the overall correlation between CEC and Apo A1 concentrations. This is an expected finding as apolipoprotein A1 standard SP1-01 was chosen as an International Reference Material (IRM) by the World Health Organization (WHO) in 1992 and has been in use since then (*41*, *42*). This led to the widespread use of fully automated immunoturbidimetric or immunonephelometric analysis systems for the simple and quick evaluation of Apo A1. These efforts were also reflected in the results of this analysis, as most studies included in this meta-analysis used one of these methods for the quantification of Apo A1. New methods exist for quantifying HDL particle numbers in human serum or plasma samples. Nuclear magnetic resonance (NMR) spectroscopy for HDL particle quantitation has already been used in clinical trials and introduced to the clinical laboratory as automated analysis systems (*43*, *44*). Nuclear magnetic resonance spectroscopy allows for accurate HDL particle number quantification, rather than approximate particle number evaluation by Apo A1 (*7*). In addition, NMR allows the evaluation of HDL particle size while keeping the original sample unchanged. These qualities of the NMR method may help to explore the link between HDL particle subpopulations and their impact on the measurement of CEC in more depth (*45*). However, NMR-based HDL particle number estimation methods lack standardization and have not yet been evaluated and approved for clinical relevance and use. Due to these reasons, it is advisable to stick to the current standardized Apo A1 measurement methods while evaluating CEC.

The authors would also like to highlight the limitations of this study. One of the main limitations of the current study is the predominantly male pooled population. The higher prevalence of male participants in the pooled sample greatly impacts the generalizability of the study results to a wider scope and highlights the well-known problem of female sex underrepresentation in the biomedical field. Another limitation is that the age of participants in the analysis was not included. As the scope of this analysis was to explore the correlation between two HDL biomarkers, CEC and Apo A1 concentrations, and various methodological aspects that may influence this measure, we chose not to include participants’ ages. Nonetheless, exploring whether age impacts the association between these two biomarkers would be worthwhile. Another limitation is the evaluation of study quality. No standardized study quality evaluation questionnaires or scales are tailored specifically to clinical chemistry or laboratory medicine fields, and correlational analysis. Thus, the authors decided to use a self-developed study evaluation tool tailored specifically to the methodological aspects of CEC studies and correlational analysis. Using such an evaluation tool may have impacted the evaluation of study quality. Lastly, this meta-analysis observed high heterogeneity among the included studies. This heterogeneity was partly explained by using different cell lines and cholesterol acceptors in the studies. However, there may have been other factors that contributed to the overall high heterogeneity, which were not evaluated in the current analysis. Such factors may have been other methodological aspects of CEC or Apo A1 analyses and factors related to the study sample sizes, participant ethnicities, and lifestyles.

## Data Availability

The dataset used and/or analyzed during the current study is available from the corresponding author on reasonable request.
